# Prevalence of Medication-Related Osteonecrosis of the Jaw in Patients with Breast Cancer, Prostate Cancer, and Multiple Myeloma

**DOI:** 10.3390/dj4040032

**Published:** 2016-09-27

**Authors:** Petra Rugani, Christian Walter, Barbara Kirnbauer, Stephan Acham, Yvonne Begus-Nahrman, Norbert Jakse

**Affiliations:** 1 Divison of Oral Surgery and Orthodontics, Medical University of Graz, 8010 Graz, Austria; barbara.kirnbauer@medunigraz.at (B.K.); stephan.acham@medunigraz.at (S.A.); norbert.jakse@medunigraz.at (N.J.); 2 Oral and Maxillofacial Surgery of the Mediplus Clinic, 55128 Mainz, Germany; walter@mainz-mkg.de; 3 Konzept Pharma Service GmbH, 31084 Freden, Germany; y.begus-nahrmann@konzept-pharma-service.de

**Keywords:** osteonecrosis, bisphosphonate, denosumab, multiple myeloma, breast cancer, prostate cancer

## Abstract

Medication-related osteonecrosis of the jaw is a known side-effect of antiresorptive therapy in patients with malignant diseases. Nevertheless, the exact pathogenesis is still unknown and published prevalences show a significant range. The aim of the presented paper was to assess the prevalence of osteonecrosis (ONJ) in breast cancer, prostate cancer, and multiple myeloma patients receiving parenteral antiresorptive therapy. For this reason a PubMed search was performed and 69 matching articles comprising 29,437 patients were included in the analysis. Nine-hundred fifty-one cases of jaw necrosis were described. The overall ONJ-prevalence was 2.09% in the breast cancer group, 3.8% in the prostate cancer group, and 5.16% for multiple myeloma patients.

## 1. Introduction

In 2003 Robert E. Marx reported on 36 cases of non-healing painful bone exposure in the mandible, maxilla, or both, that were unresponsive to surgical or medical treatments in patients treated with bisphosphonates as a growing epidemic [1].

This causal association was cautiously denied in a letter by Tarassof and Csermak representing Novartis, given the fact that no such reports had occurred in multiple, well-controlled clinical trials of more than 3000 cancer patients that had been conducted as far back as the early 1990s [2].

To date jaw necrosis linked to antiresorptive therapy is an accepted side effect and no longer a matter of discussion.

In 2007 bisphosphonate-related osteonecrosis of the jaw (BRONJ) has been primarily defined as a condition of exposed, necrotic bone in the maxillofacial region that has persisted for more than eight weeks in patients who have received or are receiving treatment with bisphosphonates and have no history of radiation therapy of the jaws [3]. This definition relies on a combination of various symptoms in combination with the patient’s anamnesis. Even though the condition was firstly described over ten years ago, the underlying principles of pathogenesis are not conclusively clarified yet. Common theories deal with reduced bone remodeling, impairment of local vascularization, and neoangiogenesis [4], accumulation of microcracks in devitalized bone with empty osteocyte lacunae [5], and infection of bone via osteoclast-independent bone resorption [6,7].

Three stages were specified, the main symptom being frank bone exposure in the maxillofacial region [8]. In addition, an “at-risk category” was defined for patients who have been treated with either oral or intravenous bisphosphonates, but show no apparent exposed/necrotic bone.

As experience grew in this condition, clinicians recognized that bisphosphonate-exposed patients can show clinical and radiological signs potentially indicating osteonecrosis lacking the cardinal symptom of exposed bone in the maxillofacial region. Therefore, the AAOMS updated its classification and added a stage 0 category, implying patients who show nonspecific clinical and radiological findings or symptoms that possibly correlate to osteonecrosis. These findings must not be explicable by other conditions [3]. As a study by Fedele et al. showed that patients who present BRONJ stage 0 may be at high risk of developing advanced stages of BRONJ [9], the relevance of an early diagnosis increased. It has to be mentioned that the AAOMS classification of 2009 categorized bone exposure via fistulas stage 0, implying that no invasive treatment is needed in such cases. This was revised in 2013, when each bony exposure fulfilling the criteria was counted as evident bone necrosis, no matter the extent. Furthermore, the term BRONJ was changed to MRONJ (medication-related osteonecrosis of the jaw) taking into account other pharmaceuticals that might cause jaw necrosis besides bisphosphonates.

Consequently, published prevalences of BRONJ were steadily increasing [10,11,12,13] since this update from 2009 and 2013, augmented by MRONJ cases due to therapy with Denosumab, or antiangiogenic agents like Bevazicumab or Sunitinib.

In general the incidence rate is higher in patients with intravenous administration of bisphosphonates compared to the oral route of administration. The estimates of MRONJ for intravenous application range from around 1% [14,15] to 21% for sub-clienteles [16], but even after more than 10 years since this condition was reported there is still some lack of knowledge. The main reasons therefore are probably:

Many patients are asymptomatic for a long time and, thus, may not be diagnosed or do not relate their oral symptoms to the antiresorptive therapy.

People suffering from MRONJ have various underlying diseases. They present a heterogeneous group of patients treated by independent medical specialists. A standardized oral investigation of patients at risk has still not been implemented to date. As a consequence, the definite prevalence might be higher, as demonstrated by Walter et al. [16], who showed that prevalence of BRONJ is underestimated if thorough inspection of the oral cavity is omitted.

The condition is influenced by several risk factors, such as drug potency, type of administration, as well as individual local and systemic conditions. In many cases patients receive multiple agents that interfere with bone metabolism and may, therefore, cause or benefit the development of osteonecrosis.

## 2. Objectives

To review published prevalences of ONJ in patients with the primary diseases of breast cancer, prostate cancer, and multiple myeloma, receiving parenteral antiresorptive therapy (bisphosphonates, denosumab).

## 3. Methods

We performed a PubMed literature search using the terms: “osteonecrosis”, “incidence”, “prevalence”, “bisphosphonate”, and “denosumab”.

Inclusion criteria were: the type of primary cancer: breast cancer, multiple myeloma, or prostate cancer; parenteral antiresorptive therapy with bisphosphonate or denosumab; and a size of patient collective of at least 10 patients.

Exclusion criteria were: review papers; duplicated articles; integrated analyses of already reported studies; and animal studies.

The reports were screened for prevalence rates, and data concerning primary disease and used antiresorptive therapy were collected.

Data analysis was performed concerning the following questions:

- What is the prevalence of osteonecrosis (ONJ) relating to primary disease?
- What are the prevalences before and after the adaptions of the AAOMS classification in 2009?

To assess the overall ONJ prevalence patients were grouped according to primary disease. Thus, positive ONJ cases were weighted against the total number of patients of all included studies.

## 4. Results

One-hundred forty-one articles were found. Fifty-eight papers were excluded right away after screening of abstracts because of improper article type (review papers, case reports, prevalence not reported) or inappropriate primary disease (osteoporosis, rheumatoid arthritis, giant cell carcinoma, other cancer types).

Fifteen further manuscripts were excluded after studying the full papers. The reasons therefore were:

- Study population not described in sufficient detail;
- Primary disease for each ONJ case not assignable;
- Oral administration route;
- Double reports;
- Combined analysis of other trials;
- Estimation of prevalence; and
- Special risk situation due to performed intervention.

Sixty-eight reports comprising 29,437 patients were included in the analysis (Table 1).

Amongst those, 41 were published before 2009 (13,059 patients) and 27 since 2010 (16,378 patients). Of the total patients, 16,632 suffered from breast cancer, 4236 from prostate cancer, and 8569 from multiple myeloma.

Nine-hundred fifty-one cases of jaw necrosis were described. Amongst those, 348 had breast cancer, 161 had prostate cancer, and 442 suffered from multiple myeloma (Table 2).

Thus, the overall ONJ-prevalence was 2.09% in the breast cancer group, 3.8% in the prostate cancer group, and 5.16% for multiple myeloma patients.

**Figure 1:**
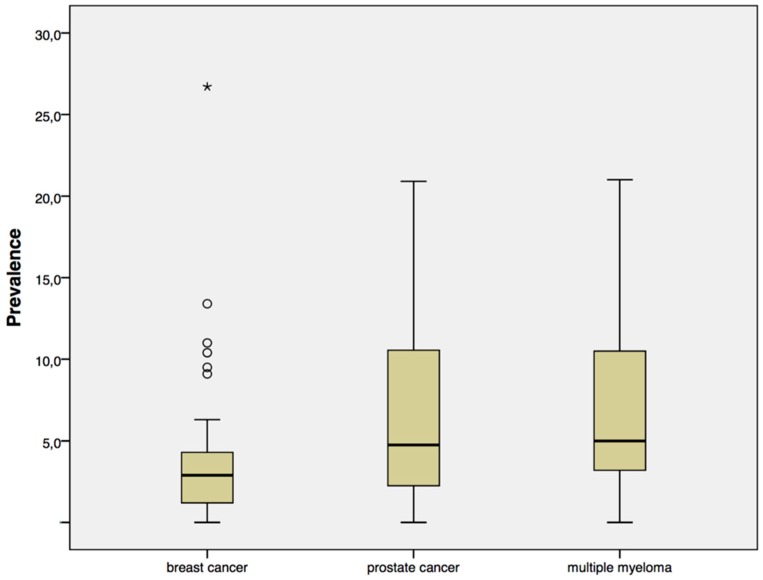
Published prevalences.

Published prevalences in patients with breast cancer range from 0 to 26.7% (median 2.6%), from 0 to 20.9% (median 4.8%) in prostate cancer patients, and from 0 to 20.5% (median 5.1%) in patients with multiple myeloma (Figure 1).

**Figure 2:**
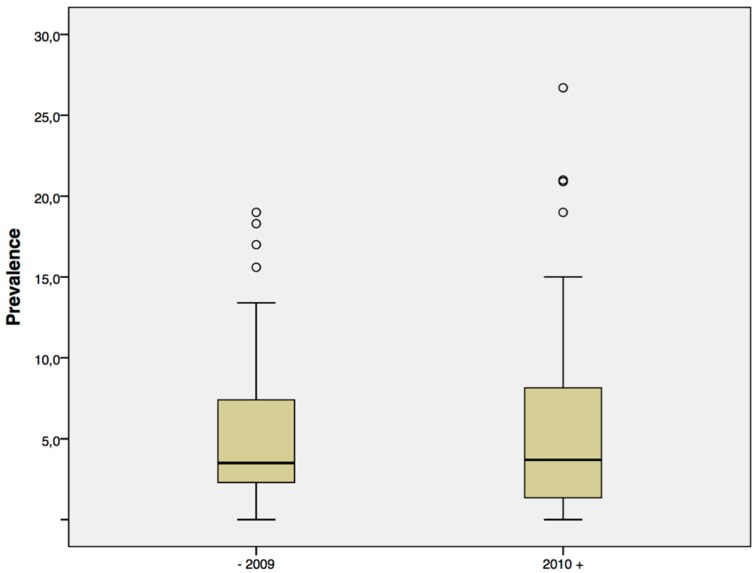
Published prevalences in reports until 2009 and since 2010.

Overall prevalence in all patients published until 2009 ranges from 0 to 18.6% (median 3.6%) and from 0 to 26.7% (median 3.7%) in papers published since 2010 (Figure 2).

## 5. Discussion

In this paper we determined the weighted prevalence of medication-related osteonecrosis of the jaw in patients with breast cancer, prostate cancer, and multiple myeloma, including 69 reports comprising 29,670 patients. Prevalence was 2.09% in the breast cancer group, 3.8% in the prostate cancer group, and 5.16% for multiple myeloma patients.

To the best of our knowledge there are three further reports addressing this issue. In 2009 Walter et al. [46] provided an overview including 25 studies from 2005 to 2008 comprising 5825 patients and reported on lower prevalences in breast cancer patients compared to prostate cancer and multiple myeloma patients. In 2010 Walter et al. [16] also addressed the problem of possible under-reporting of MRONJ cases if dental examinations are left out. In 2014 Boquete-Castro et al. published a systemic analysis evaluating the adverse advents of Denosumab [82]. They found seven articles reporting on 97 MRONJ cases. Incidences were low and ranged between 0 and 2%, but exceeded the incidences in the zoledronate control group. Published prevalences of medication-related osteonecrosis of the jaw show a great range. Possible explanations have been discussed in the literature and may include variations in sample size, varying methods of data assessment/analysis, and limitations due to voluntary case reporting [65]. Furthermore, when evaluating the published literature we had to realize that in most papers MRONJ definition is not clearly stated. Very often descriptions are not detailed and do not refer to a specific classification system, or are even left out completely.

Despite this inhomogeneity it has been shown that the application of zoledronic acid produces a greater risk than the use of pamidronate or the combination of zoledronate and pamidronate [25]. Additionally, ONJ onset seems to be earlier among patients receiving zoledronic acid as compared to patients receiving pamidronate [18], and even later if alendronate or risedronate is used [25,83,84].

Regarding denosumab, ONJ prevalence was slightly higher compared with zoledronate, but no significant difference was found [70].

In general, ONJ develops more frequently if parenteral administration of antiresorptive agents are used [85,86,87] and significantly correlates with the number of applied doses [12,53,65]. Differences in applied dosing protocols may also be the reason for the discrepancy of ONJ prevalences in patients with different primary diseases, resulting in a higher risk for patients suffering from multiple myeloma (weighted ONJ-prevalence 5.16%) compared to patients with breast cancer (weighted ONJ prevalence 2.09%).

Beyond that several additional factors influencing the risk of MRONJ have been identified.

Concomitant oral disease, respectively, oral inflammation, may increase the risk of ONJ [14,67], as well as wearing of dentures [47] and anatomical circumstances. ONJ occurs more frequently in the mandible with described predilection for the molar and premolar region, as well as regions with thin mucosal coverage, like tori or the mylohyoid line [70,87,88].

Oral surgery, and above all, dental extractions, are most frequently named to increase ONJ risk around seven- to more than ten-fold [14,20,53,70,89].

Taylor et al., who reviewed the clinical records of 225 patients at risk who underwent dental extraction, identified eight ONJ cases out of 23 (34.8%) patients prescribed intravenous bisphosphonates and five ONJ cases in 202 patients (2.5%) with oral bisphosphonate administration [90]. However, the periodontal and inflammation status in the surrounding bone might be a more important factor in the development of osteonecrosis rather than the procedure of the extraction itself [91].

In addition, systemic risk factors have been described. ONJ onset seems to be more frequent in women and in advanced age [17,86,87,88]. This might be attributed to the fact that important primary diseases, like breast cancer and osteoporosis, are more frequent in women, respectively, and that cancer and osteoporosis commonly develop with increasing age. Regarding the group of multiple myeloma patients, no difference in gender could be detected [20,25,50,87].

Furthermore, the wearing of removable mucosa-retained dentures also becomes more likely with increasing age and is a known risk factor. Patients with removable dentures are more likely to develop osteonecrosis compared to patients with fixed partial dentures and patients without substitution of potentially messing teeth [92].

Concomitant medical therapy may enhance ONJ risk, which has been particularly reported for corticosteroids and antiangiogenic therapy. A correlation to chemotherapy, diabetes, or anaemia is less substantiated [12,20,25,70], although a recently published article sees an association to a pathological glucose metabolism [93]. Altogether, systemic risk factors seem to have less impact than local variables [70].

Finally, differences in diagnostic criteria, mainly resulting from differences or alterations in classification guidelines, may also have an impact, albeit we could not detect a difference in published prevalences until 2009 compared to those since 2010, after the revision of AAOMS guidelines. It could be hypothesized that intensifying oral inspections to detect early forms (Stage 0) and possibly including patients presenting sinus tracts into verified ONJ cases, resulted in a higher prevalence as already demonstrated [63]. However, this effect might be compensated by the greater awareness towards ONJ risk and, consequently, the implementation of preventive measures. Routine dental prevention to eliminate potential sites of infection prior to antiresorptive treatment and managing risk factors in patients at risk reduces the BRONJ rate, as already described in the literature [12,50,52,94,95]. Furthermore, it has been reported that dental extraction and oral surgery are quite safe if certain rules are obeyed [13,15,96,97].

## 6. Conclusions

The exact pathogenic mechanisms of MRONJ development are still unknown, but risk factors have been described. Patients with malignant diseases carry a relevant risk for MRONJ. It has been shown that it is possible to reduce ONJ risk by implementation of routine dental preventive measures and management of known risk factors. Consequently, a close cooperation between medical specialties involved in the treatment of malignoma and multiple myeloma patients at risk is crucial to minimize ONJ prevalence.

## Figures and Tables

**Table 1:** Included studies.

Year	Author	Study Design	Disease	Patients (n)	ONJ Cases	Prevalence (%)	Used Agent
2005	Bamias [17]	pros	breast ca	70	2	2.86	Z, PZ, ZI
		pros	mult myel	111	11	9.91	Z, PZ, ZI
		pros	prostate ca	46	3	6.52	Z, PZ, ZI
	Durie [18]	web survey	mult myel	904	62	6.86	Z, P
		web survey	breast ca	299	13	4.35	
	Guarneri [19]	retro	breast ca	48	3	6.25	P
2006	Badros [20]	retro	mult myel	340	11	3.24	P, Z, PZ
	Calvo-Villas [21]	retro	mult myel	64	7	10.94	Z
	Dimopoulos [22]	pros	mult myel	202	15	7.43	Z
	Sanna [23]	pros	breast ca	81	5	6.17	P, Z
	Tosi [24]	retro	mult myel	259	9	3.47	Z
	Zervas [25]	pros	mult myel	254	28	11.02	Z, P, ZP
	Ortega [26]	?	breast ca	126	2	1.59	Z
2007	Aguiar Bujanda [27]	css	breast ca	35	4	11.43	Z
	Corso [28]	retro	mult myel	106	8	7.55	Z, PZ
	García Sáenz [29]	pros	prostate ca	104	3	2.88	Z
	Jadu [30]	retro	mult myel	655	21	3.21	P
	Ortega [31]	retro	prostate ca	52	6	11.54	Z
	Petrucci [32]	?	mult myel	311	22	7.07	Z, P, PZ
	Wang [33]	retro	mult myel	292	11	3.77	Z, P, Z
		retro	breast ca	81	2	2.47	Z, P, Z
		retro	prostate ca	69	2	2.9	Z, P, Z
	Lipton [34]	prosp	breast ca	211	0	0	D
	Pozzi [35]	retro	mult myel	1402	28	2	Z, PZ
2008	Boonyapakorn [36]	pros	mult myel	58	10	17.24	P, PZ, IZ, Z
	Fehm [37]	retro	breast ca	233	10	4.29	Z, ICPZ
	Ibrahim [38]	retro	breast ca	220	5	2.27	PZ, Z
		retro	mult myel	59	2	3.39	PZ, Z
	Walter [39]	css	prostate ca	43	8	18.6	IZ, PZ, Z
	Yonemori [40]	prosp	breast ca	18	0	0	D
	Ellis [41]	prosp	breast ca	106	0	0	D
	Christodoulou [42]	retro	breast ca	75	2	2.67	Z, I
		retro	prostate ca	11	1	9.1	Z, I
	Estilo [43]	retro	breast ca	134	18	13.43	P, Z, PZ
		retro	prostate ca	31	4	12.9	P, Z, PZ
		retro	mult myel	145	6	4.14	P, Z, PZ
	Hoff [14]	retro	breast ca	1338	16	1.2	P, Z
		retro	mult myel	548	13	2..37	P, Z
	Montefusco [44]	retro	mult myel	178	9	5.06	BP
	Musto [45]	prosp	mult myel	81	1	1.23	Z
2009	Walter [46]	css	breast ca	75	4	5.33	Z, PZI
	Aragon-Ching [47]	pros	prostate ca	60	11	18.33	Z
	Cetiner [48]	pros	mult myel	32	5	15.63	Z
	Crawford [49]	retro	breast ca	113	10	3.5	P, PZ, Z
	Dimopoulos [50]	pros	mult myel	128	16	12.5	Z
	Haidar [51]	retro	prostate ca	51	2	3.92	Z
	Ripamonti [52]	retro	breast ca	590	18	3.05	P, PZ, Z
		prosp	breast ca	112	2	1.79	P, PZ, Z
	Vahtsevanos [53]	retro	breast ca	1041	32	3.07	Z, P, I, PZ, IZ
		retro	mult myel	539	46	8.53	P, PZ, Z
		retro	prostate ca	41	2	4.88	P, Z, ZI
	Fizazi [54]	prosp	prostate	17	0	0	P, Z
		prosp	breast ca	16	0	0	P, Z
		prosp	prostate ca	33	0	0	D
		prosp	breast ca	30	0	0	D
	Bonomi [55]	retro	breast ca	238	7	2.94	P, PZ, Z
		retro	protate ca	46	1	2.17	P, PZ, Z
	Stumpe [56]	retro	mult myel	128	3	2.34	P, Z, PZ
		retro	breast ca	241	1	0.41	P, Z, PZ
		retro	prostate ca	128	1	0.78	P, Z, PZ
2010	Walter [16]	retro	mult myel	81	4	4.94	U, PZ
		css	mult myel	78	16	20.51	Z, PZ, IZ, PZI
	Bantis [11]	retro	prostate ca	60	9	15	Z
	Gimsing [57]	retro	breast ca	250	8	3.2	P normal dose (90 mg)
		retro	breast ca	252	2	0.79	P low dose (30 mg)
	Pakovic [58]	retro	mult myel	190	2	1.05	P, PI, I
	Stopeck [59]	prosp	breast ca	1020	20	1.96	D
		prosp	breast ca	1013	14	1.38	Z
2011	Fizazi [60]	prosp	prostate ca	950	22	2.32	D
		prosp	prostate ca	951	12	1.26	Z
	Quispe [61]	retro	breast ca	110	10	9.09	Z
2012	Ding [62]	retro	breast ca	181	1	0.55	P, I, Z
	Miyazaki [63]	retro	prostate ca	111	9	8.11	Z
	Smith [64]	prosp	prostate ca	716	33	4.61	D
	Thumbigere-Math [65]	retro	breast ca	190	8	4.21	P, PZ, Z
		retro	mult myel	83	6	7.23	P, PZ, Z
		retro	prostate ca	84	2	2.38	P, PZ, Z
	Rugani [66]	retro	breast ca	48	5	10.42	Z
	Then [67]	retro	mult myel	120	23	19.17	P, Z, I
	Martin [68]	prosp	breast ca	1026	0	0	D
	Henry [69] + Saad [70]	prosp	mult myel	180	6	3.33	D,Z
2013	Assaf [71]	retro	breast ca	95	9	9.47	P, I, Z, ZI, PI
		retro	mult myel	42	5	11.9	P, I, Z, ZI, PI
	Coleman [72]	prosp	breast ca	1065	5	0.47	Z
	Brufsky [73]	retro	breast ca	159	6	3.77	P, Z, PZ
		retro	breast ca	62	1	1.61	P, Z, PZ
	Rathbone [74]	prosp	breast ca	1681	26	1.55	Z
2014	Barrett-Lee [75]	prosp	breast ca	697	9	1.29	Z
	Coleman [76]	prosp	breast ca	1685	26	1.54	Z
	Jackson [77]	prosp	mult myel	981	36	3.67	Z
	Gnant [78]	prosp	breast ca	900	0	0	Z
2015	Vidal-Real [79]	retro	prostate ca	43	9	20.93	Z
		retro	breast ca	15	4	26.67	Z
		retro	mult myel	18	0	0	Z
	Rodrigues [80]	prosp	prostate ca	324	2	0.62	Z
2016	Stopeck [81]	prosp	breast ca	318	20	6.29	D
		prosp	breast ca	334	18	5.39	ZD
		prosp	prostate ca	147	12	8.16	D
		prosp	prostate ca	118	7	5.93	ZD

Abbreviations: prosp (prospective), retro (retrospective), ccs (cross-sectional study), ? (unknown), ca (cancer), mult myel (multiple myeloma), Z (zoldronate), P (pamidonate), I (ibandronate), D (denosumab).

**Table 2:** Weighted prevalences in breast cancer, prostate cancer, and multiple myeloma patients.

	Breast Cancer			Prostate Cancer			Multiple Myeloma			Total		
	n	Cases	prev	n	Cases	prev	n	Cases	prev	n	Cases	prev
−2009	5531	156	2.82%	732	44	6.01%	6796	344	5.06%	13,059	544	4.17%
2010+	11,101	192	1.73%	3504	117	3.34%	1773	98	5.53%	16,378	407	2.49%
Total	16,632	348	2.09%	4236	161	3.80%	8569	442	5.16%	29,437	951	3.23%

Abbreviations: n (number of patients); prev (prevalence); Cases (number of patients with ONJ).
